# Electronic Structure of Rock Salt Alloys of Rare Earth and Group III Nitrides

**DOI:** 10.3390/ma13214997

**Published:** 2020-11-06

**Authors:** Maciej J. Winiarski

**Affiliations:** Institute of Low Temperature and Structure Research, Polish Academy of Sciences, Okólna 2, 50-422 Wrocław, Poland; m.winiarski@int.pan.wroc.pl

**Keywords:** nitride semiconductors, band gap, calculations

## Abstract

Lattice parameters and electronic properties of $RE_{1-x}A_{x}$N alloys, where *RE* = Sc, Y, Lu and *A* = Al, Ga, and In, have been derived from first principles. The materials are expected to exhibit a linear decrease in cubic lattice parameters and a tendency to a linear increase in band gaps as a function of composition. These effects are connected with a strong mismatch between ionic radii of the *RE* and group III elements, which leads to chemical pressure in the mixed *RE* and group III nitrides. The electronic structures of such systems are complex, i.e., some contributions of the *d*- and *p*-type states, coming from *RE* and *A* ions, respectively, are present in their valence band regions. The findings discussed in this work may encourage further experimental efforts of band gap engineering in *RE*-based nitrides via doping with group III elements.

## 1. Introduction

Rare earth (*RE*) nitrides (ScN, YN, and LuN) are semiconductors with indirect band gaps ($E_{g}^{\Gamma -X}$) in a range from 0.9 to 1.3 eV [1,2,3,4,5,6]. Their ground state phase is the rock salt structure in contrast to the zinc-blende phase, which is generally adopted by III-V semiconductors and wurtzite structure characteristic for group III nitrides [7].

Numerous applications of group III nitride materials in optoelectronics caused interest in structural and electronic properties of novel Ga${}_{1-x}$Sc${}_{x}$N [8,9,10], Al${}_{1-x}$Sc${}_{x}$N [11,12,13], and Al${}_{1-x}$Y${}_{x}$N [14] alloys. Despite the fact that the high crystalline quality of some wurtzite thin films of Ga${}_{1-x}$Sc${}_{x}$N was suggested for a wide range of contents [8], such materials were obtained for x<0.3 in the following studies [9,10]. Furthermore, the rock salt phase in mixed *RE* and group III nitrides was suggested as very robust in the recent theoretical investigations [15].

Wurtzite solid solutions of group III and rare earth nitrides exhibit a linear dependence of $E_{g}$ on a *RE* content [8,9,10,11,12,13,14,16,17,18]. Namely, an introduction of Sc and Y ions in such systems leads to a reduction of $E_{g}$ with respect to those of the parent AlN and GaN compounds. Similar effect was also reported for rock salt Al${}_{1-x}$Sc${}_{x}$N [19]. In turn, solid solutions of *RE*N semiconductors are expected to exhibit strong bowings of $E_{g}$, which are related to a significant variation in radii of $RE^{3+}$ ions in these materials [20,21].

In this work, the electronic structure of rock salt alloys of *RE* and group III nitrides was investigated within the methods of the density functional theory (DFT). The predicted lattice parameters and band gaps of semiconducting nitride alloys are presented as a function of their composition. Effects of introduction of group III ions into rock salt ScN, YN, and LuN materials on their electronic structures are discussed based on the structural properties, density of states, band gaps, and findings of available experimental reports.

## 2. Results and Discussion

The cubic lattice parameters of rock salt unit cells of the nitrides, obtained within the LDA approach, are gathered in Table 1. The available experimental data for *RE*N and AlN indicate slightly larger volumes of the unit cells of these materials than in our results. The rock salt GaN and InN were only obtained under high pressure and thus were not included into the table [22,23].

It is worth mentioning that the ionic radii of *RE* elements (except Sc) are generally larger than those of their group III counterparts. One may consider that the relatively small mismatch between unit cell volumes of certain compounds indicates rather feasible formation of their heterogeneous solid solutions, whereas other systems, e.g., Lu${}_{1-x}$Al${}_{x}$N and Y${}_{1-x}$Al${}_{x}$N, are expected to be difficult to obtain in a wide composition range. The assumption that the content of group III ions in the materials studied here is less than 0.5 can be considered justified as it is consistent with the findings of the experimental reports [8,9,10,11,12,13,14] and the recent theoretical predictions [15].

**Figure 1:**
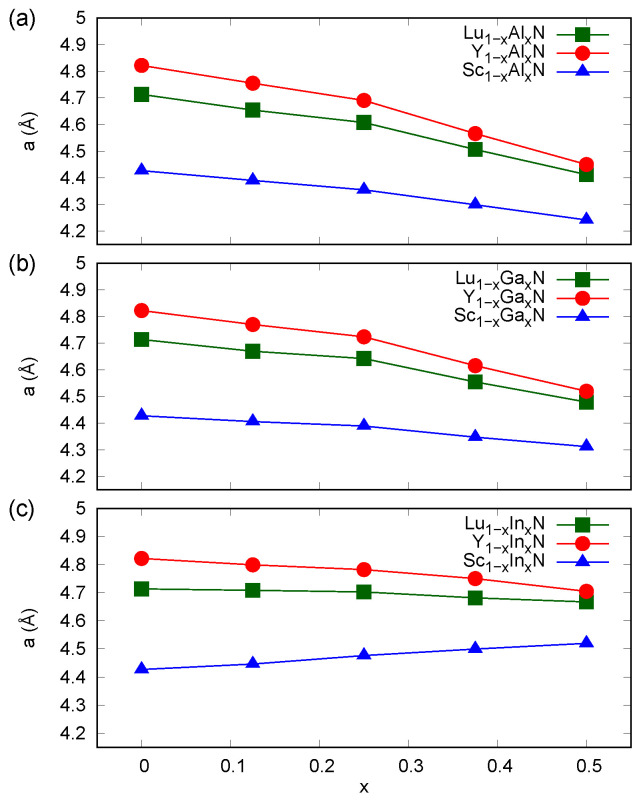
Cubic lattice parameters calculated (LDA) for rock salt alloys (**a**) $RE_{1-x}$Al${}_{x}$N, (**b**) $RE_{1-x}$Ga${}_{x}$N, (**c**) $RE_{1-x}$In${}_{x}$N, where *RE* = Sc, Y, and Lu.

The lattice parameters of solid solutions of *RE* and group III nitrides are presented in Figure 1. As may be expected, the introduction of relatively smaller ions into parent *RE*N systems leads to a decrease in lattice parameters of the resulting materials. The Sc${}_{1-x}$In${}_{x}$N alloys are the only exceptions. The dependences *a*(*x*) are close to linear. The deviations from this character are evident in systems that contains ions of strongly different radii. It is worth recalling that similar effects were reported for ternary *RE*N alloys [20,21], as well as Al${}_{1-x}$In${}_{x}$N nitrides [29]. In turn, the $RE_{1-x}$In${}_{x}$N materials exhibit almost ideal linear dependences of lattice parameters on the indium content.

**Figure 2:**
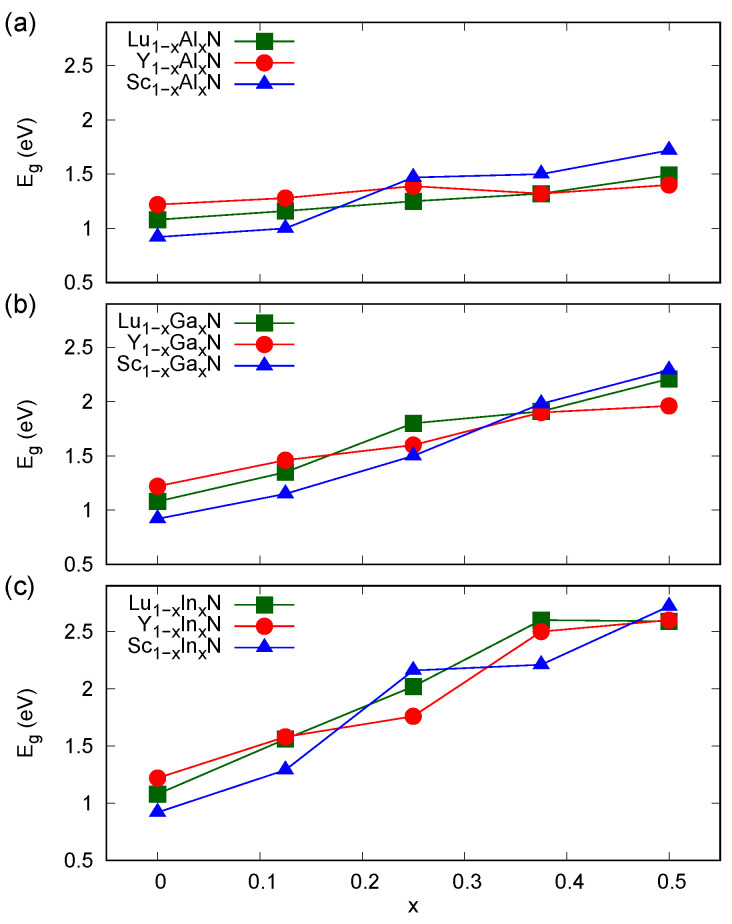
Band gaps calculated (MBJLDA) for rock salt alloys (**a**) $RE_{1-x}$Al${}_{x}$N, (**b**) $RE_{1-x}$Ga${}_{x}$N, (**c**) $RE_{1-x}$In${}_{x}$N, where *RE* = Sc, Y, and Lu.

Regarding the band gaps of the solid solutions considered here, as depicted in Figure 2, their dependences on a composition are also of a linear type. This finding comes from experimental studies of certain wurtzite and rock salt *RE*-based materials [8,9,10,11,12,13,14] and is opposite to a tendency of formation of strong band gap bowings, characteristic for group III nitrides [29]. The band gaps of rock salt group III nitrides of 6.33, 2.83, and 1.48 eV, calculated here for AlN, GaN, and InN, respectively, are bigger than those of the *RE*N host materials. One may therefore expect relatively increased band gaps in the solid solutions. Interestingly, the increase in $E_{g}$ as a function of a composition is clearly connected with the ionic radius of the particular group III ion. A relatively small change in $E_{g}$ was revealed for the $RE_{1-x}$Al${}_{x}$N, whereas the corresponding effect in the $RE_{1-x}$In${}_{x}$N alloys is very strong. This phenomenon is connected with a strong influence of the tensile strain on $E_{g}$ in *RE*N systems, discussed in [6]. The *RE*N materials with decreased volumes of unit cell are expected to exhibit smaller $E_{g}$. The presence of Al ions, in which the *d*-type electronic shells are unoccupied, is equivalent to chemical pressure in the neighboring region of a material. In turn, the In ions are heavier and exhibit fully occupied *d*-type bands, and affect the electronic structure of a particular system to a greater extent.

**Figure 3:**
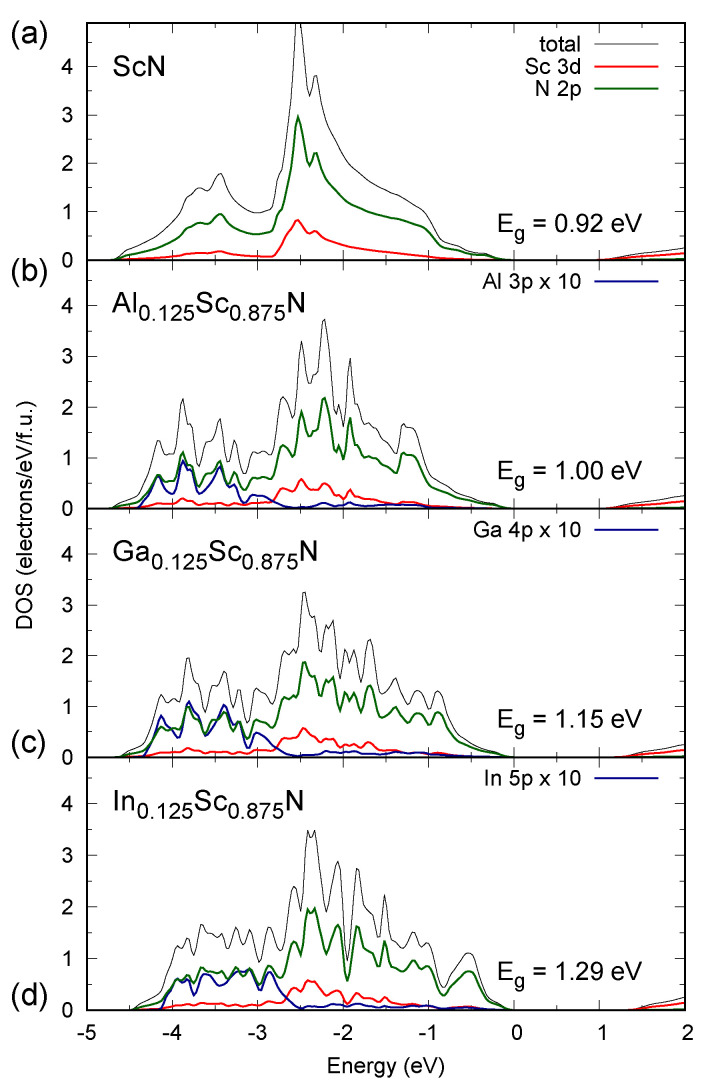
Density of states calculated (MBJLDA) for rock salt alloys (**a**) ScN, (**b**) Al${}_{0.125}$Sc${}_{0.875}$N, (**c**) Ga${}_{0.125}$Sc${}_{0.875}$N, and (**d**) In${}_{0.125}$Sc${}_{0.875}$N. Please note that the *p*-type contributions of group III ions are magnified by 10.

**Figure 4:**
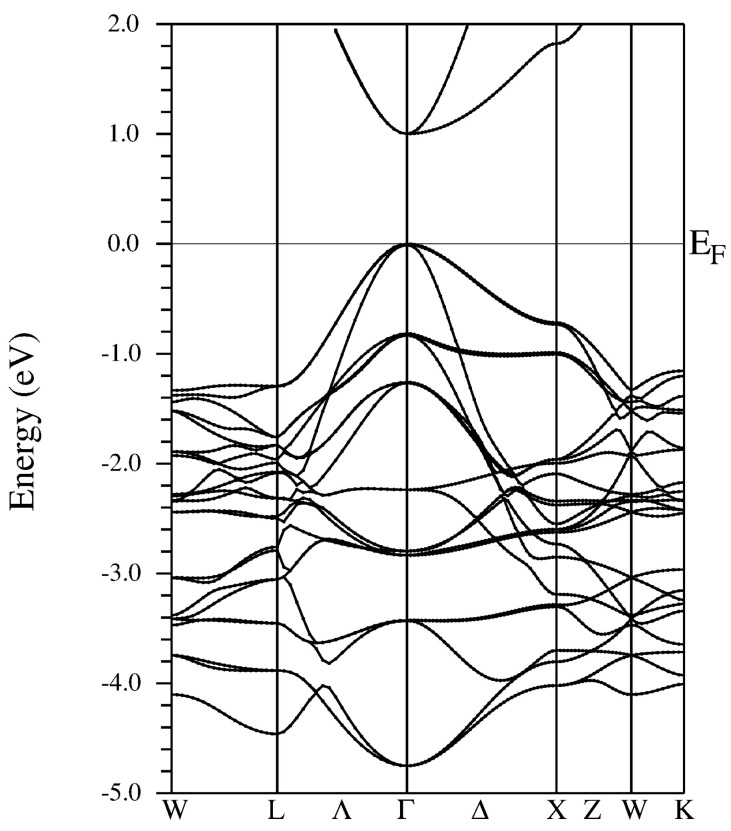
Band structure calculated (MBJLDA) for rock salt Al${}_{0.125}$Sc${}_{0.875}$N material.

As presented for Sc-based systems in the density of states (DOS) plots in Figure 3, the electronic structure of the valence regions of the materials studied in this work are dominated by the 2p contributions coming from nitrogen ions, which is a characteristic feature of nitrides in general. The valence band maximum (VBM) is also formed by the *d*-type states of *RE* ions. It is worth noting that the *p*-type and *s*-type (not shown in Figure 3) bands of group III ions are located well below VBM, i.e., in the energy region below −2.5 eV. As depicted for Al${}_{0.125}$Sc${}_{0.875}$N in Figure 4, these bands are also pronounced in a band structure. Their shapes are relatively flat, which results in gap regions in some directions in the Brillouin zone (the *W* – *L*, *L* – Γ – *X*, and *X*–*W*–*K* lines). Furthermore, the overall shapes of DOS of the systems, depicted in Figure 3, are very similar to each other. The presence of Al/Ga/In dopant ions does not affect VBM of the ScN host material, which is an opposite effect to one reported for group III nitride alloys [29]. This feature is common for all materials considered in this study. The findings presented here are consistent with the above discussion of possible strain effects on the electronic structure of mixed *RE* and group III nitrides. They seem to be a main origin of an increase in $E_{g}$ as a function of the composition of particular systems.

## 3. Conclusions

The results of ab initio calculations indicate the doping with group III ions (Al, Ga, and In) as a reasonable strategy of band gap engineering in *RE*N materials. The enlargement of their band gaps may be expected with the increasing content of group III ions, which is related to their ionic radii. Such a doping does not lead to significant changes in the valence band regions of the materials. The most significant effects are found for $RE_{1-x}$In${}_{x}$N systems, which are expected to be the most feasible to be obtained in experimental efforts. The findings presented in this work may encourage further experimental studies concerning the electronic structure of *RE* and group III nitride alloys.

## 4. Materials and Methods

Equilibrium geometries of *RE*N materials were studied with the use of the Abinit package [30,31], i.e., the lattice parameters and atomic positions of the rock salt 2 × 2 × 2 supercells (the multiplication of a primitive cell, 16 atoms) of ternary *RE*N alloys were found via stresses/forces relaxation. The PAW atomic datasets taken from the JTH table [32] with the Perdew-Wang [33] (LDA) parameterization of the exchange-correlation energy were employed in this task. The valence basis sets were Al: $3s^{2}3p^{1}$, Ga: $4s^{2}4p^{1}3d^{10}$, In: $5s^{2}5p^{1}4d^{10}$, Sc: $3s^{2}3p^{6}4s^{2}3d^{1}$, Y: $4s^{2}4p^{6}5s^{2}4d^{1}$, Lu: $5s^{2}5p^{6}6s^{2}5d^{1}4f^{14}$, and N: $2s^{2}2p^{3}$. Next, the Wien2k package [34] was used for calculations of fully relativistic MBJLDA [35] band structures. Such a complex calculation process employs a cost-effective pseudopotential approach for full structural optimizations (the stress tensor) and highly accurate calculations of electronic structures with the full potential method. The standard convergence criteria for total energy and forces, and the 12 × 12 × 12 **k**-point meshes were used in all calculations. The energy plane-wave basis cutoff of 15 Ha and $RK_{max}$ of 8 were employed in pseudopotential and full potential calculations, respectively.

## Figures and Tables

**Table 1:** Lattice parameters calculated in this work ($a_{LDA}$), available experimental data ($a_{exp}$ [24,25,26,27]) for rock salt nitrides, and ionic (3+) radii of the rare earth and group III elements [28].

Compound	$a_{\mathrm{LDA}}$ (Å)	$a_{\exp}$ (Å)	IR (pm)
AlN	4.030	4.043	67.5
GaN	4.187	–	76.0
ScN	4.427	4.501	88.5
InN	4.586	–	94.0
LuN	4.714	4.766	100.1
YN	4.822	4.877	104.0
